# Alpha-Defensin 1: An Emerging Periodontitis Biomarker

**DOI:** 10.3390/diagnostics13132143

**Published:** 2023-06-22

**Authors:** Jisuk Lee, Dong Sik Chang, Junsu Kim, Young Sun Hwang

**Affiliations:** 1 Department of Biomedical Laboratory Science, College of Health Science, Eulji University, Seongnam 13135, Republic of Korea; 2 Department of Otorhinolaryngology, Eulji University Hospital, Eulji University, Daejeon 35233, Republic of Korea; 3 Seoul Hana Dental Clinic, Seongnam 13636, Republic of Korea; 4 Department of Dental Hygiene, College of Health Science, Eulji University, Seongnam 13135, Republic of Korea

**Keywords:** alpha-defensin 1, periodontitis biomarker, inflammation, gingival crevicular fluids, neutrophile, osteoclast

## Abstract

*Background*: Research on the development of reliable diagnostic targets is being conducted to overcome the high prevalence and difficulty in managing periodontitis. However, despite the development of various periodontitis target markers, their practical application has been limited due to poor diagnostic accuracy. In this study, we present an improved periodontitis diagnostic target and explore its role in periodontitis. *Methods*: Gingival crevicular fluid (GCF) was collected from healthy individuals and periodontitis patients, and proteomic analysis was performed. The target marker levels for periodontitis were quantified in GCF samples by enzyme-linked immunosorbent assay (ELISA). Mouse bone marrow-derived macrophages (BMMs) were used for the osteoclast formation assay. *Results*: LC-MS/MS analysis of whole GCF showed that the level of alpha-defensin 1 (DEFA-1) was higher in periodontitis GCF than in healthy GCF. The comparison of periodontitis target proteins galactin-10, ODAM, and azurocidin proposed in other studies found that the difference in DEFA-1 levels was the largest between healthy and periodontitis GCF, and periodontitis was more effectively distinguished. The differentiation of RANKL-induced BMMs into osteoclasts was significantly reduced by recombinant DEFA-1 (rDEFA-1). *Conclusions*: These results suggest the regulatory role of DEFA-1 in the periodontitis process and the relevance of DEFA-1 as a diagnostic target for periodontitis.

## 1. Introduction

Although early-onset periodontitis can be restored to normal gums with appropriate treatment, the treatment prognosis is poor because most cases are diagnosed as chronic [1]. The World Health Organization (WHO) Global Oral Health Status Report estimated that approximately 19% of the adult population worldwide has severe periodontal disease (https://www.who.int/news-room/fact-sheets/detail/oral-health (accessed on 20 January 2023); Oral health, WHO, 2022). Periodontitis tends to be diagnosed as chronic because the initial symptoms are usually mild and diagnosed by a dentist. Therefore, the development of diagnostic tools for the effective management of periodontitis is actively progressing, and the development of target markers with high diagnostic accuracy is needed. Several diagnostic markers have been developed [2,3]. However, their diagnostic accuracy is poor, limiting their practical use.

Samples were analyzed for saliva, oral microbes, oral metabolites, saliva occult blood, and gingival crevicular fluid (GCF) to develop target markers for periodontitis [3]. Saliva is easy to collect but is less suitable because of contamination with oral microorganisms and food debris. Microbial cultures and genomic analysis are required for detecting oral microorganisms in samples, presenting limitations to the development of target markers. In addition, detecting metabolites in the oral cavity using the commonly used immune response-based diagnostic kits is not easy. The method of analyzing occult blood in saliva is not suitable for diagnosing early periodontitis without bleeding. Unlike saliva, GCF contains various components, such as cellular elements (epithelial cells, leukocytes, and bacteria), electrolytes, minerals, organic compounds, immunoglobulins, complement, cytokines (interleukin-1 and tumor necrosis factor alpha), metabolic products, and enzymes released from blood vessels around the periodontium, and its components change according to sensitive changes in the environment around the periodontal pocket [4]. Since GCF can be collected noninvasively with absorbent paper, it is an ideal specimen for use in the search for target markers for periodontitis. This study reviewed the development process of periodontitis diagnostic markers using GCF as a sample and confirmed that most of the target markers were identified by various mass chromatograms after protein separation, such as electrophoresis [5,6,7,8]. Acrylamide gel electrophoresis and two-dimensional (2D) electrophoresis are limited to the analysis of proteins of 10 kilodaltons or less. Therefore, low-molecular-weight proteins or peptides were not reflected in the development process of the previously presented diagnostic markers for periodontitis. GCF is rich in low-molecular-weight proteins secreted from blood vessels around the periodontium. Therefore, this study speculates that the inadequacy of the sample analysis process in the search for diagnostic markers for periodontitis also limited the development of targets with high diagnostic accuracy.

Alpha-defensins (DEFAs) are a major family of host defense peptides with a mass of 4–10 kilodaltons, and are particularly abundant in neutrophils, certain macrophage populations, and the Paneth cells of the small intestine [9]. Defensins are constitutively expressed but are also produced in response to microbial products or pro-inflammatory cytokines. Neutrophil-derived DEFA can enhance phagocytosis by human and murine macrophages [10]. In addition, DEFA amplifies the local inflammatory response by increasing the production of pro-inflammatory factors (interleukin-1, tumor necrosis factor, histamine, and prostaglandin D2) by monocytes. In this study, whole GCF was analyzed by LC-MS/MS for the first time to search for periodontitis diagnostic targets that could improve diagnostic accuracy. Through this, it was confirmed that the low-molecular-weight protein DEFA-1 (alpha-defensin 1) was increased in periodontitis GCF. Thus, we investigated the relevance of DEFA-1 as a diagnostic target for periodontitis.

## 2. Material and Methods

### 2.1. Reagents

All chemicals used in the experiments were of analytical grade and were purchased from Sigma-Aldrich (St. Louis, MO, USA). Cell culture medium was purchased from Gibco (Gibco-BRL, Grand Island, NY, USA). Anti-DEFA-1 and β-actin were purchased from Novus (Novus Biologicals, Centennial, CO, USA). Recombinant human receptor activator of nuclear factor kappa-B ligand (RANKL) and recombinant human macrophage colony-stimulating factor (M-CSF) were purchased from R&D System (Minneapolis, MN, USA).

### 2.2. Study Population and GCF Preparation

Voluntary participant recruitment and all clinical procedures were performed by a dentist (JS Kim) at the Seoul Hana Dental Clinic (Seongnam, Republic of Korea). The participants were provided with oral and written information about the study and then signed an informed consent form prior to enrollment. Adults with at least 22 erupting teeth were included as subjects. People with oral mucosal inflammation and pregnant women were excluded from the study. GCF samples were collected from the mediolabial and distolabial regions of maxillary first and second premolars with periodontitis. The GCF collection site was dried with an air syringe, and saliva contamination was minimized with a cotton swab. Paperpoint (Meta Biomed) was carefully inserted into the periodontal pocket of individuals with healthy periodontal tissue (n = 85) and patients with periodontitis (n = 130), and GCF was collected for 30 s. Thereafter, the absorbent paper was immersed in phosphate-buffered saline (PBS) in an Eppendorf tube and kept for one hour. After centrifugation at 3000× *g* for 5 min at 4 °C to remove undissolved materials, the supernatant was collected and stored at −80 °C until analysis. The sample size was calculated using G*Power sampling software (version 3.1.9.7 for Windows) with an α error of 5% and power of 95% and was determined to be 52 per group. Additional samples were collected from each group to compensate for processing errors. The gender ratio and age of the study subjects by group are provided in Table 1. The healthy group consisted of subjects with no evidence of clinical attachment loss, clinical inflammation, fissure hemorrhage, or radiographic bone loss. The periodontitis group consisted of participants whose teeth had a probing depth greater than 3 mm and clinical attachment loss greater than 3 mm. The plaque index (PI) and gingival index (GI) were measured based on the criteria of Silness and Löe [11]. This study was conducted in accordance with the Declaration of Helsinki and was approved by the Institutional Review Board of Eulji University (EU19-62).

### 2.3. GCF Proteome Analysis

GCF collected from healthy individuals and periodontitis patients was pooled and subjected to proteomic analysis. A total of 25 µg protein from the pooled GCF was used for proteomic analysis, and all sample preparation procedures for protein precipitation, peptide digestion, and fractionation were analyzed with a Q Exactive HF-X hybrid quadrupole-Orbitrap mass spectrometer. Peptide mass analysis was performed according to a previous method [12]. Data analysis was performed using Proteome Discover 2.4 software (Thermo Fisher, Waltham, MA, USA) for protein identification and label-free quantification. SEQUEST-HT, part of the Proteome Discoverer 2.4 software, was used for database searching against the UniProt human database. GCF proteomic analysis was conducted at the Proteomics Core Facility at the National Cancer Center (Goyang, Republic of Korea).

### 2.4. Western Blot Analysis

Fifty micrograms of pooled GCF was separated by 15% SDS-PAGE. For Western blot analysis, proteins separated in polyacrylamide gels were transferred to polyvinylidene difluoride (PVDF) membranes (Millipore). Membranes were blocked with 5% skim milk and reacted with anti-DEFA-1 (1:1000 dilution) overnight at 4 °C. Membranes were then reacted with the respective HRP-conjugated secondary antibodies (1:3000 dilution), and DEFA-1 protein was detected using an enhanced chemiluminescence (ECL) detection kit. Using the Image J program, we compared DEFA-1 levels in healthy GCF and periodontitis GCF.

### 2.5. Enzyme-Linked Immunosorbent Assay (ELISA)

An ELISA was performed to quantify the target protein in the GCF. The following ELISA kits were purchased from their respective sources: human DEFA-1 ELISA kit (DY8198-05) (R&D System), human azurocidin ELISA kit (ab213755) (Abcam, Cambridge, UK), human ODAM ELISA kit (A1E959) (Cusabio Biotech, Wuhan, China), and human Galectin-10 ELISA kit (NBP2-75319) (Novus Biologicals, Centennial, CO, USA). The analysis was performed according to the manufacturers’ methods. Each GCF sample was reacted in a microplate well pre-coated with a clonal antibody specific for the target protein. After brief washing, enzyme-conjugated secondary polyclonal antibody and substrate solutions were added to the wells and reacted according to the protocol. After the color change after 30 min was checked, stop solution was added, and absorbance was measured at 450 nm in a microplate reader (Synergy TM multimode microplate reader, BioTek Instruments Inc., Winooski, VT, USA). The target protein concentration in the GCF was calculated in nanograms per milliliter using a standard curve.

### 2.6. Osteoclast Formation Assay

Density gradient centrifugation using Histopaque was used to isolate mouse bone marrow-derived macrophages (BMMs) from the tibiae of BalbC *nu/nu* mice (female, 4 weeks old). The isolated BMMs were directly used for the osteoclast formation assay in α-MEM supplemented with 10% FBS and 30 ng/mL M-CSF (216-MC, R&D system, Minneapolis, MN, USA) in a humidified atmosphere at 37 °C and 5% CO_2_. Osteoclast formation was induced by 100 ng/mL RANKL treatment (390-TN, R&D system). To confirm the effect of DEFA-1 on osteoclast formation, recombinant DEFA-1 protein (ab9934, Abcam) was added to the medium. BMMs were cultured with fresh medium every 2 days. After 5 days, cells were fixed with paraformaldehyde, and osteoclasts were identified by staining with tartrate-resistant acid phosphatase (TRAP) using the Acid Phosphatase Leukocyte kit (Sigma-Aldrich, St. Louis, MO, USA). TRAP-positive multinucleated (≥3 nuclei) cells were counted by light microscopy. The experiment was approved by the Animal Ethics Committee of Eulji University (approval number: EUIACUC22-13).

### 2.7. Statistical Analysis

GraphPad Prism ver. 5.01 statistical software was used for statistical analyses. The Mann–Whitney test was used to analyze the data between the periodontitis and healthy groups. The Kruskal–Wallis ANOVA with Dunn’s post hoc analysis was used for the multiple comparisons. The results are presented as mean ± SD. *p* values lower than 0.05 were considered statistically significant.

## 3. Results

### 3.1. DEFA-1 Levels Increase in Periodontitis GCF

This study analyzed GCF from 85 participants with healthy gums and 130 participants with periodontitis. The healthy participants consisted of 30 males and 55 females (mean age, 38.94 ± 14.47 years). The periodontitis group consisted of 87 males and 43 females (mean age, 50.19 ± 22.15 years). The PI and GI were measured on four surfaces of the teeth (buccal gingival margin, distal buccal gingiva, mesial buccal, and lingual surfaces). The PI and GI of the periodontitis group (mean PI, 1.76 ± 0.51, mean GI, 1.80 ± 0.38) were significantly higher than those of the healthy group (mean PI, 0.12 ± 0.21, mean GI, 0.05 ± 0.45) (Table 1).

**Figure 1:**
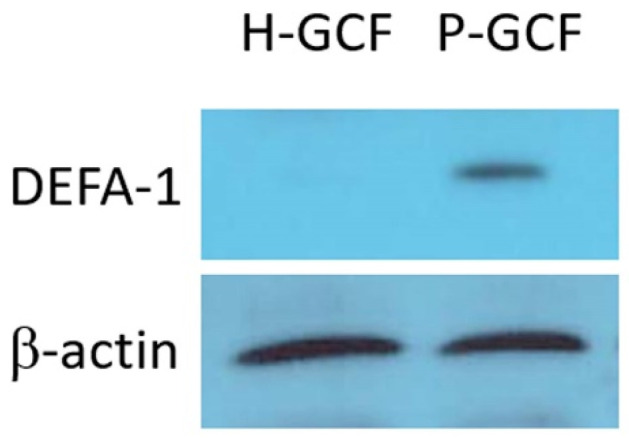
Western blot analysis for DEFA-1. Twenty-five micrograms of protein from a GCF sample from healthy subjects (H-GCF) and patients with periodontitis (P-GCF) was separated on 15% acrylamide gel electrophoresis, and a Western blot was performed with DEFA-1-specific primary antibody.

Healthy GCF (H-GCFs, n = 85) and periodontitis GCF (P-GCFs, n = 130) were pooled to provide sufficient protein for proteomic analysis. Pooled H-GCF and P-GCF were prepared by collecting equal volumes from each of the GCF samples. As a result of searching the UniProt human database using SEQUEST-HT with a protein identification criterion of at least two unique peptides per protein, a total of 1422 proteins were identified by analyzing 25 µg of GCF protein by LC-MS/MS (Supplementary Data). Among them, 1295 proteins were commonly detected in H-GCF and P-GCF. The average spectral number of 589 proteins was increased by more than 2-fold in P-GCF compared to H-GCF and decreased by more than 2-fold for 138 proteins. LC-MS/MS analysis showed that the abundance of DEFA-1 in P-GCF was 2.743-fold higher than that of DEFA-1 in H-GCF (*p* = 0.048) (Table 2). In the proteomic analysis, DEFA-1 was the only protein detected in GCF among the defensin superfamily. A total of 25 µg of GCF protein was analyzed by Western blotting using a DEFA1-specific antibody, and a higher DEFA-1 protein level was observed in P-GCF compared to H-GCF (Figure 1).

### 3.2. DEFA-1 Quantification in Healthy GCF and Periodontitis GCF

**Figure 2:**
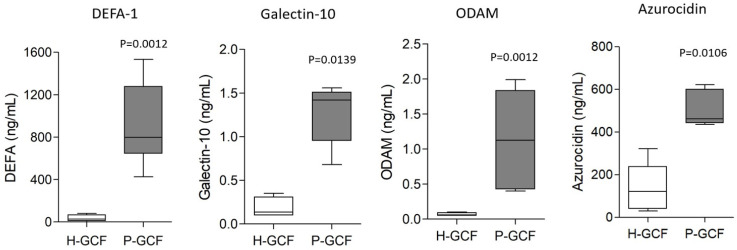
ELISA analysis for DEFA-1, galectin-10, ODAM, and azurocidin. Concentrations of target proteins were quantified in GCF from healthy subjects (H-GCF, n = 85) and patients with periodontitis (P-GCF, n = 130). *p* value vs. H-GCF.

DEFA-1 concentration in GCF was measured by ELISA analysis. As shown in Figure 2, the DEFA-1 concentration in P-GCF (mean, 911.37 ng/mL ± 391.05) was significantly higher than that of the H-GCF group (mean, 35.54 ng/mL ± 30.64) (*p* = 0.0012). ELISA analysis of the target proteins previously suggested as diagnostic markers for periodontitis was performed with the same GCF sample. The concentration of galectin-10 in P-GCF (mean, 1.23 ng/mL ± 0.33) was significantly higher than that in H-GCF (mean, 0.181 ng/mL ± 0.12) (*p* = 0.0139). The concentration of odontogenic ameloblast-associated protein (ODAM) in P-GCF (mean, 1.18 ng/mL ± 0.74) was also significantly higher than that in H-GCF (mean, 0.07 ng/mL ± 0.02) (*p* = 0.0012). However, the concentrations of galectin-10 and ODAM in GCF were relatively lower than those of DEFA-1. The concentration of azurocidin in P-GCF (mean, 509.01 ng/mL ± 79.43) was significantly higher than that in H-GCF (mean, 142.15 ng/mL ± 118.31) (*p* = 0.0106). However, despite a significant difference in azurocidin concentration in P-GCF compared to H-GCF, the concentration of azurocidin in H-GCF (mean, 142.15 ng/mL) was significantly higher than that of the other analyzed targets (DEFA-1 mean, 35.54 ng/mL; galectin-10 mean, 0.181 ng/mL; ODAM mean, 0.07 ng/mL).

### 3.3. Inhibitory Effect of DEFA-1 in Osteoclast Formation

**Figure 3:**
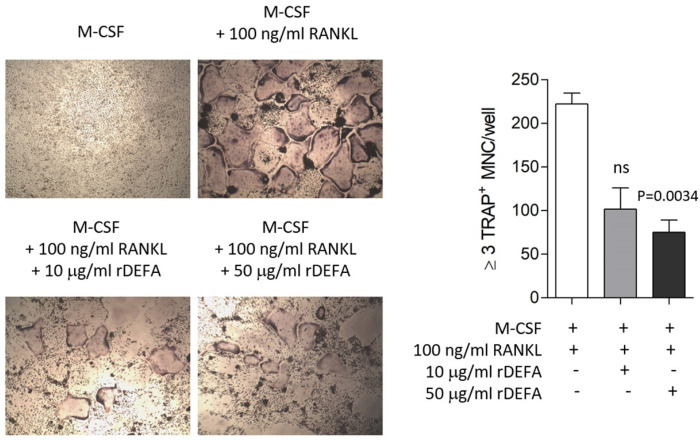
Effect of recombinant DEFA-1 protein (rDEFA) on RANKL-induced osteoclastic bone resorption. BMM cells were treated with M-CSF (30 ng/mL) and RANKL (100 ng/mL) with or without rDEFA (10 or 50 µg/mL) for 5 days and were stained to detect the expression of TRAP. The total number of TRAP-positive multinucleated (≥3 nuclei) osteoclasts (MNCs) per well is graphically presented. The data are expressed as the mean ± SD. *p* vs. M-CSF + RANKL condition. ns = non-specific.

An osteoclast formation assay was performed to confirm the role of DEFA-1 during periodontitis progression. BMMs isolated from mouse tibiae were differentiated into osteoclasts in a medium containing macrophage colony-stimulating factor (M-CSF) (30 ng/mL) and RANKL (100 ng/mL). The effect of DEFA-1 on the differentiation process of BMMs into osteoclasts was observed by adding recombinant DEFA-1 protein. Compared to the number of osteoclasts produced in M-CSF and RANKL media, the addition of recombinant DEFA-1 (rDEFA-1) protein significantly reduced osteoclast formation, as shown in Figure 3. The inhibition of osteoclast formation by the addition of 50 µg/mL rDEFA-1 was statistically significant (*p* = 0.0034).

## 4. Discussion

In vitro diagnostic tests are useful to both dentists and patients as an auxiliary diagnostic tool for periodontitis. A noninvasive periodontal diagnosis is effective in managing the patient’s long-term periodontal health. In particular, the need for in vitro diagnostic tests for periodontal conditions, such as peri-implantitis, increases as the number of dental implant placement cases increases. Active research is being conducted to develop diagnostic biomarkers useful for diagnosing periodontitis [5,6,7,8]. GCF in the periodontal pocket contains components related to defense mechanisms for controlling the inflammation of gingival tissue and is involved in maintaining the homeostasis of the periodontal pocket [3]. The diagnostic biomarkers for periodontitis suggested by GCF analysis include superoxide dismutase (SOD1), apolipoprotein A-I (ApoA-I), dermcidin (DCD), L-plastin, annexin-1, azurocidin, and ODAM [5,6,7,8,9,10,11,12,13,14,15]. Our LC-MS/MS analysis of GCFs also identified these proteins, except for DCD. The abundance ratio of each protein in H-GCF and P-GCF was as follows: SOD1 (abundance ratio vs. H-GCF: 2.217, *p* < 0.05), ApoA-I (abundance ratio vs. H-GCF: 5.724, *p* > 0.05), annexin-1 (abundance ratio vs. H-GCF: 1.292, *p* > 0.05), azurocidin (abundance ratio vs. H-GCF: 4.101, *p* < 0.05), and ODAM (abundance ratio vs. H-GCF: 1.309, *p* < 0.05). L-plastin (Plastin-1) was only detected in P-GCF. Although their use as diagnostic markers has been verified, their practical use is limited due to their low diagnostic accuracy for periodontitis. In addition, these diagnostic markers were found in searches for proteins with molecular weights high enough to be separated by acrylamide during the GCF analysis process, so low-molecular-weight proteins or peptides were excluded from the analysis. Therefore, the proteomic analysis of whole GCF is required to develop a new diagnostic target for periodontitis.

In this study, GCF was noninvasively collected using Paperpoint, and whole GCF was analyzed by LC-MS/MS to derive a new diagnostic biomarker for periodontitis targeting low-molecular-weight secreted proteins. The proteomic analysis showed that alpha-defensin 1 (DEFA-1) was the only defensin superfamily member identified in GCF, and it is the first protein suggested as a diagnostic target for periodontitis. DEFA-1 is a 10 kDa protein produced by the innate immune system and epithelial cells [9]. It is stored in neutrophilic granules but is also secreted into the extracellular fluid. When inflammation begins, the amount of exudate flowing into the periodontal pocket from the surrounding blood vessels increases. Therefore, the relatively high DEFA-1 concentration in periodontitis GCF compared to normal GCF is thought to be a result of the inflammatory response. In addition to DEFA-1, the study quantified the concentrations of target proteins presented as diagnostic markers for periodontitis in normal GCF and periodontitis GCF. The ELISA analysis showed that the concentrations of galectin-10, ODAM, and azurocidin were significantly higher in periodontitis GCF (P-GCF) compared to healthy GCF (H-GCF). However, compared to DEFA-1 (mean, 35.54~911.37 ng/mL), the protein concentrations of galectin-10 and ODAM were low (galectin-10 mean, 0.181~1.23 ng/mL; ODAM mean, 0.07~1.18 ng/mL). Therefore, a highly sensitive detection system would be needed to use galectin-10 and ODAM as diagnostic markers. Compared to DEFA-1, the azurocidin concentration was lower in P-GCF (0.56-fold, DEFA-1 (mean, 911.37 ng/mL) vs. azurocidin (mean, 509.01 ng/mL)) and higher in H-GCF (4-fold, DEFA-1 (mean, 35.54 ng/mL) vs. azurocidin (mean, 142.15 ng/mL)). Therefore, DEFA-1 was confirmed as a more suitable target to distinguish between health and periodontitis conditions than azurocidin. Galectin-10 is a 16 kDa protein that is exclusively expressed in eosinophils and basophils. Galectin-10 is degranulated and released upon stimulation of the outer membrane [16,17,18]. Azurocidin is secreted from neutrophils as a lysosomal protein and acts as a multifunctional inflammatory mediator [5]. ODAM is involved in the direct attachment of the junctional epithelium to the tooth surface and is released from the epithelium when attachment to the tooth surface is lost during periodontitis [6]. Galectin-10, azurocidin, and ODAM were all increased in GCF at the site of periodontal inflammation, suggesting them as diagnostic markers for periodontitis. Therefore, the use of multiple diagnostic markers, which include these targets together with DEFA-1, will be effective in increasing the accuracy of periodontitis diagnoses.

In this study, we performed the proteomic analysis of whole GCF to find a diagnostic target for periodontitis with improved diagnostic accuracy and confirmed that DEFA-1 was suitable for distinguishing between healthy and periodontitis conditions. Compared to healthy periodontal GCF, significantly higher DFEA-1 levels were detected in periodontitis GCF. DEFA-1 was also significantly increased in the saliva of patients with gingivitis and was suggested as a biochemical parameter for diagnosing gingivitis [19]. In addition, DEFA-1 promotes the polarization of M1 to M2 macrophages and is involved in the pathogenesis of osteoarthritis, suggesting it as a target for the diagnosis of infection after total joint arthroplasty [20,21]. A high copy number of the DEFA-1 gene was also confirmed in Danish Crohn’s disease patients [22]. These results suggest that DEFA-1 is involved in several inflammatory diseases. Unlike gingivitis, which is inflammation limited to the gums, periodontitis is a periodontal disease in which inflammation progresses to the gums, periodontal ligament, and alveolar bone. The periodontal pocket deepens as inflammation of the gingival sulcus progresses, leading to loss of the periodontal ligament and alveolar bone resorption [23,24]. Osteoclasts can be formed by the differentiation of immature cells of the monocyte–macrophage lineage, and RANKL and M-CSF secreted from osteoblasts act as essential signaling molecules for differentiation into osteoclasts. M-CSF provides cell survival signals for osteoclast progenitors and osteoclasts. The binding of RANKL into the RANK receptor of osteoclast progenitors induces differentiation into osteoclasts by activating TRAF6, NF-κB, c-fos, JNK, ERK, and PI3K pathways [25]. Human neutrophil peptides are known as α-defensins [9]. DEFA-1 is known to be involved in the innate host inflammatory defense by promoting neutrophil recruitment, enhancing the production of pro-inflammatory cytokines, and activating complement. According to the previous report, M-CSF-derived differentiation of peripheral blood monocytes into macrophages was inhibited by DEFA-1 [26]. This means that DEFA-1 plays a role in the differentiation of hematopoietic cells. Therefore, an osteoclast formation assay was performed to elucidate the role of DEFA-1 in the process of alveolar bone destruction following periodontitis progression. When the differentiation of BMM into osteoclasts was induced by RANKL, the addition of recombinant DEFA-1 protein significantly reduced osteoclast formation. This suggests that DEFA-1 suppresses osteoclast formation, according to the inflammatory response around the alveolar bone. As a result, DEFA-1 levels may be increased in periodontitis GCF compared to healthy GCF.

## 5. Conclusions

In this study, increased levels of DEFA-1 in the GCF of patients with periodontitis were confirmed by proteomics and ELISA analyses. ELISA quantitative analysis confirmed that DEFA-1 distinguished between the healthy condition and periodontitis more clearly than previously suggested periodontitis diagnostic targets such as galectin-10, azurocidin, and ODAM. Since the level of DEFA-1 in the GCF of periodontitis patients was higher than the levels of previously suggested periodontitis diagnostic targets, it will be more useful as an in vitro diagnostic tool. Therefore, our results suggest that DEFA-1 is a useful periodontitis target marker that can overcome the limitations of practical applications due to poor diagnostic accuracy. The study also demonstrated the role of DEFA-1 in the inhibition of osteoclast formation during alveolar bone resorption caused by periodontitis through in vitro experiments. Despite the anti-periodontitis role of DEFA-1, chronic and excessive inflammation will lead to periodontitis with alveolar bone resorption. Although further studies are needed to verify the usefulness of DEFA-1 as a biomarker in a large cohort divided by periodontitis severity, the detection of DEFA-1 in GCF could be very useful for the noninvasive diagnosis of periodontitis.

## Figures and Tables

**Table 1:** Distribution of study subject characteristics in the healthy and periodontitis groups.

	Healthy Group (n = 85)	Periodontitis Group (n = 130)	*p*-Value
Gender			
Male (n, (%))	30 (35.3)	87 (66.9)	0.041
Female (n, (%))	55 (64.7)	43 (33.1)	
Age (years, mean ± SD)	38.94 ± 14.47	50.19 ± 22.15	<0.05
PI (mean ± SD)	0.12 ± 0.21	1.76 ± 0.51	<0.001
GI (mean ± SD)	0.05 ± 0.45	1.80 ± 0.38	<0.001

**Table 2:** Identification of differentially expressed defensin 1 alpha (DEFA-1) in periodontitis GCF compared to healthy GCF.

Accession	Description	Reactome Pathways	Gene Symbol	MW (kDa)	Sum PEP Score	#Peptides	Abundances (H, Healthy)	Abundances (P, Periodontitis)	Fold Change (P vs. H)	*p*-Value (P vs. H)
P59665	Neutrophil defensin 1	Alpha-defensins	DEFA-1	10.2	329.868	4	49.9	136.9	2.743	0.048

## Data Availability

Data are available from the corresponding author upon reasonable request.

## Supplementary Materials

The following supporting information can be downloaded at: https://www.mdpi.com/article/10.3390/diagnostics13132143/s1, Supplementary Data: differentially expressed proteins data.
